# Sodium hypochlorite body wash in the management of *Staphylococcus aureus–*colonized moderate‐to‐severe atopic dermatitis in infants, children, and adolescents

**DOI:** 10.1111/pde.13842

**Published:** 2019-04-15

**Authors:** Sara Majewski, Tanya Bhattacharya, Manuela Asztalos, Benjamin Bohaty, Katherine C. Durham, Dennis P. West, Adelaide A. Hebert, Amy S. Paller

**Affiliations:** ^1^ Department of Dermatology Northwestern University Feinberg School of Medicine Chicago Illinois; ^2^ Department of Dermatology The UTHealth McGovern Medical School‐Houston Houston Texas

**Keywords:** atopic dermatitis, bleach, body wash, sodium hypochlorite

## Abstract

**Objectives:**

A cleansing body wash containing diluted sodium hypochlorite (0.006% NaOCl) was evaluated for management of moderate‐to‐severe *Staphylococcus aureus–*colonized, atopic dermatitis in children.

**Methods:**

A 6‐week, prospective, open‐label study was conducted with 50 evaluable participants (ages 6 months to 17 years) who had moderate‐to‐severe atopic dermatitis with *S aureus* skin colonization documented by culture. Participants were instructed to continue using their current medications while using the study product, 0.006% NaOCl body wash, once daily to affected areas for 6 weeks. Primary outcome measures were Investigator's Global Assessment, Eczema Area and Severity Index, and Body Surface Area scores. Secondary outcome measures were the Visual Analog Scale for pruritus, Family Dermatology Life Quality Index, and Patient Satisfaction Questionnaire for Problem Areas. A subject daily diary and a six‐item subject questionnaire that provided information on preferences for bleach bath vs body wash were secondary outcome measures.

**Results:**

Daily use of the 0.006% NaOCl body wash led to improvement for all outcome measures comparing baseline to 2‐week and to 6‐week evaluations. Of the 50 skin *S aureus*‐positive subjects, 32/50 (64%) were still positive at 2 weeks. A 36.5% decrease in subject's daily record of topical corticosteroid application at end of study compared to baseline was found. Participant surveys indicated preferences for the body wash over bleach baths.

**Conclusions:**

Sodium hypochlorite (NaOCl) body wash improved all outcome measures for moderate‐to‐severe *S aureus*–colonized AD in infants, children, and adolescents. The limited reduction in *S aureus* further suggests that sodium hypochlorite has ameliorative effects other than antimicrobial actions.

## 1 INTRODUCTION

Up to 90% of patients with atopic dermatitis (AD) have colonization by *Staphylococcus aureus* (SA) of lesional skin and often nares and nonlesional skin, even when overt clinical signs of infection are lacking.^1^, ^2^, ^3^, ^4^, ^5^, ^6^ SA has been shown to promote skin inflammation and exacerbate AD. Furthermore, topical application of commensal organisms with anti‐SA activity suppresses AD inflammation.^7^, ^8^

The purported role of SA in exacerbating AD was a major impetus for the original use of 0.005%‐0.006% NaOCl (dilute bleach), considered a bactericidal agent with very low potential toxicity.^9^, ^10^, ^11^, ^12^ Early studies provided evidence that dilute NaOCl improved AD severity in children with moderate‐to‐severe AD.^9^, ^10^ In a 3‐month trial, twice‐weekly bleach baths plus intranasal mupirocin led to greater reduction in AD severity and body surface area involvement than plain water baths plus intranasal petrolatum in 31 children with moderate‐to‐severe AD who had an initial skin infection treated with cephalexin.^10^, ^11^, ^13^ The greater effect of dilute NaOCl in bleach baths at 1 month and 3 months was seen only in submerged body parts, suggesting the requirement for exposure to the treated bathwater for efficacy. Wong et al^9^ evaluated 36 moderate‐to‐severe AD subjects without overt clinical skin infection in a 2‐month randomized, double‐blinded, placebo‐controlled trial and also reported improved disease severity with the use of twice‐weekly dilute bleach baths. Two shorter studies and a meta‐analysis based on only the first 4 weeks of therapy have disputed the effect of dilute NaOCl; however, given that bleach baths are primarily used as maintenance therapy, 4 weeks may be an insufficient time to effect significant change.^14^, ^15^, ^16^ Despite a small increase in pH by adding bleach to water, no reduction in skin barrier function based on skin hydration, pH, and tewametry has been shown with use of bleach baths for AD.^17^

While submersion in bleach baths is a commonly used approach to managing AD in children, a bathtub is not always an option and bathing may not be as convenient as showering. A NaOCl body wash may be a viable alternative for those who require and/or prefer showering. Daily use of a commercially available 0.006% NaOCl body wash (CLn^**®**^ Body Wash, Top MD Skin Care Inc., Dallas, TX) in a 12‐week, open‐label trial of 18 children with moderate‐to‐severe AD led to reduced Investigator's Global Assessment (IGA; *P* = 0.001), diminished affected body surface area (BSA; *P* = 0.005), and significantly improved parent‐reported outcomes (*P* < 0.001) and satisfaction with body wash use.^18^ To further assess the daily use of 0.006% NaOCl body wash in AD management, we conducted a larger, two‐center trial in pediatric patients with moderate‐to‐severe AD and laboratory evidence of SA colonization.

## 2 SUBJECTS AND METHODS

### 2.1 Study population

Subjects were recruited from the outpatient pediatric dermatology clinics at two large urban medical centers (Ann and Robert H. Lurie Children's Hospital, Chicago, IL and the UTHealth McGovern Medical School‐Houston, Houston, TX). Inclusion criteria were (a) age greater than 6 months and less than 18 years; (b) moderate‐to‐severe AD (IGA score of 3, 4, or 5); (c) ability to shower; (d) positive SA skin culture at the screening visit; and (e) one target lesion suitable for photography. Exclusion criteria included (a) active clinical skin infection (defined as heavy oozing, pus drainage, abscess, boil, cellulitis), which, in the investigator's opinion, would require systemic antibiotics; (b) use of systemic or topical antibiotic, systemic corticosteroids, or other immunosuppressive medication in the 4 weeks prior to screening; (c) any form of topical NaOCl (bleach) use in the 2 weeks prior to screening; and (d) inability to maintain all current medication(s) and topical regimen at time of screening for the subsequent 6 weeks of the study.

### 2.2 Study design

We conducted a 6‐week, prospective, sequentially enrolled, open‐label, multicenter study in accordance with Good Clinical Practice and the Declaration of Helsinki. The study was approved by the Institutional Review Board at each participating institution (ClinicalTrials.Gov:NCT01714245). Written, informed consent (and assent for children 12 years of age and older) was obtained prior to any research procedures. Baseline assessments were performed, and eligible subjects were provided with the commercially available study product containing 0.006% NaOCl body wash in a sealed, child‐safe pump dispenser to use daily for 6 weeks by wetting the body thoroughly with showering, lathering the study product on the body with special attention to AD‐affected areas, leaving on the skin for 2 minutes, and rinsing off with warm water. Participants were provided diaries to document continuation of their existing baseline AD regimen, including topical corticosteroids, but subjects were to refrain from adding new topical treatments. Systemic or topical antibiotics, systemic corticosteroids, immunosuppressive medications, and traditional bleach baths or leave‐on NaOCl‐containing agents were not allowed during the study period.

### 2.3 Assessments

#### 2.3.1 Efficacy assessments

Efficacy assessments were performed at all visits (baseline, 2 weeks, and 6 weeks) by a study physician, and continuity for assessments was maintained throughout the study. Primary end points were IGA, EASI,^19^ and BSA scores. Investigator Global Assessment (IGA) is an overall assessment performed with a 6‐point scale ranging from 0 (clear) to 5 (very severe). Body surface area (BSA) was assessed using the palmar method,^20^ in which the subject's “handprint” area, including the fingers and palm, is equivalent to 1% of the total body surface area. Secondary end points included the Visual Analog Scale (VAS) for pruritus,^21^ Children's Dermatology Life Quality Index (CDLQI; for ages 4 years and above),^22^ and Family Dermatology Life Quality Index (FDLQI).^23^ In addition, a subject daily diary related to study product usage, topical corticosteroid usage, topical nonsteroidal usage, and topical nonprescription usage were collected for assessment. Also, a six‐item subject questionnaire administered at each of three study visits queried the subjects about bleach bath vs the study product.

#### 2.3.2 Bacteriological assessments

Nonquantitative bacterial cultures (sample collected by rubbing a moist swab over the affected area) and polymerase chain reaction (sample collected using the modified scrub technique)^24^ were performed on a target lesion at baseline and at 2 weeks to document SA colonization. The percentage of subjects positive for SA was a secondary end point of the study. *Staphylococcus aureus* sensitivity testing was performed at baseline and at 2 weeks to determine if methicillin‐sensitive (MSSA) or methicillin‐resistant (MRSA).

#### 2.3.3 Other collected information

Photography of AD‐involved skin was performed at each visit. Diaries of concurrent AD regimen usage, including topical corticosteroids, were collected and assessed while using the NaOCl body wash. Parents, along with age‐appropriate subjects, completed a questionnaire at each visit asking them to rate the NaOCl body wash from 1 to 10 (10 is best) with regard to their experience with use of the study product and their opinion on recommendation for future use.

#### 2.3.4 Safety assessments

Subjects and parents were queried about adverse events (AE) at each visit for documentation and investigator evaluation. Parents reported AEs for those subjects who were not age appropriate for communication on their own behalf.

### 2.4 Statistical analyses

Statistical analyses included two‐sided paired *t* tests. The intention‐to‐treat population of 62 enrolled subjects was analyzed for safety. Subjects who failed screening (prior to the baseline visit) were not included in the analyses for outcomes. For subjects withdrawn from the study after the baseline visit, the latest scoring available, either baseline or 2 weeks, was carried forward. The clinical severity score at baseline, 2 weeks, and 6 weeks and the relative reduction from baseline to 2 weeks, as well as baseline to 6 weeks, were calculated as means.

## 3 RESULTS

**Figure 1:**
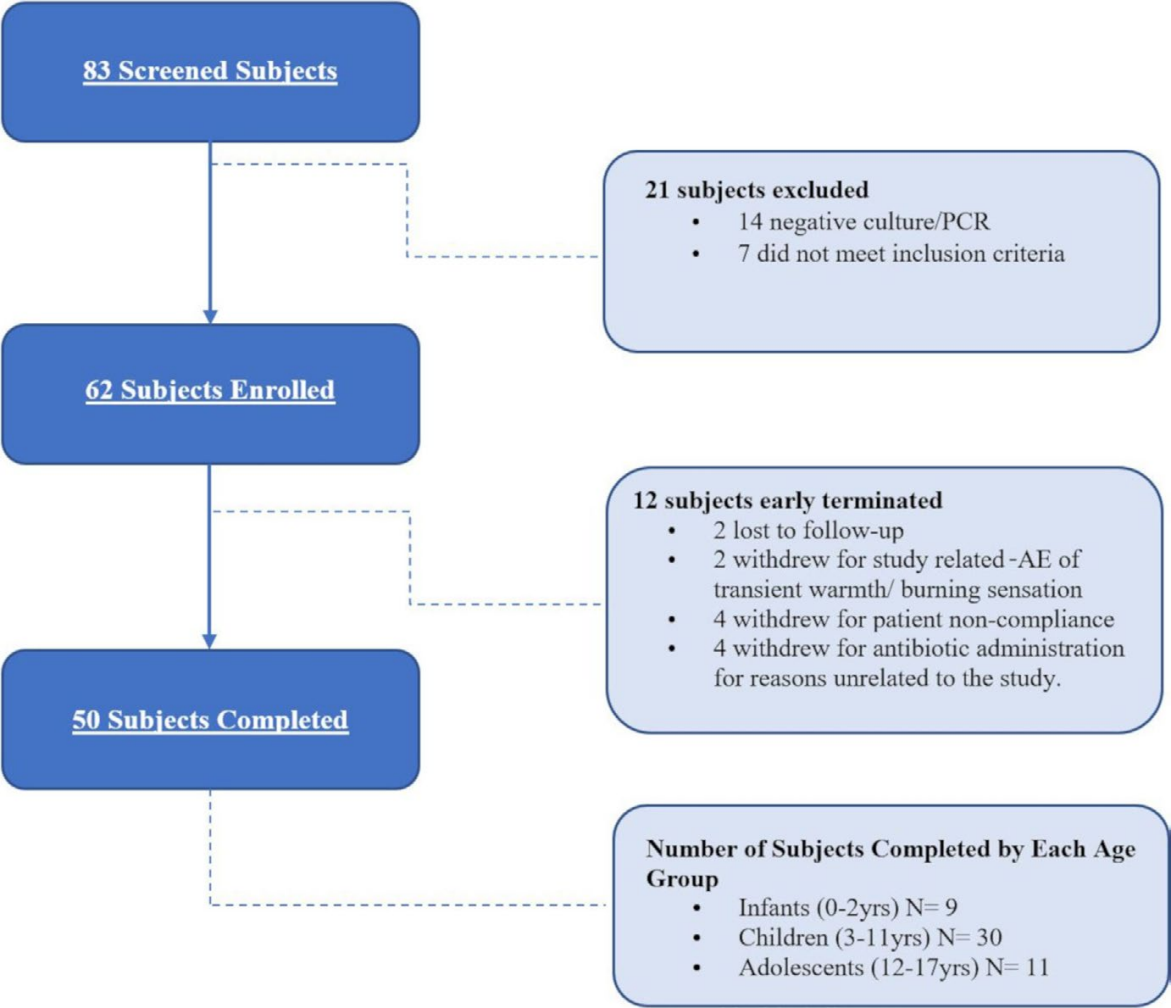
Study enrollment reflecting the intent‐to‐treat population

Subjects were enrolled and completed the study within a 12‐month period. Eighty‐three subjects were screened (Figure 1). Of the 83‐screened participants, 21 were excluded: 14 were culture‐negative, and 7 failed to meet entry criteria. Of the remaining 62 subjects who advanced to baseline and use of the NaOCl body wash, 12 terminated early, which included 6 who withdrew due to AEs (2 transient warmth or burning sensation at application site and 4 due to antibiotics started for unrelated reasons), 4 who were noncompliant, and 2 lost to follow‐up. Fifty subjects completed the study. Table 1 summarizes baseline demographic data.

**Table 1:** Baseline demographic data

Characteristics (total n = 50)	Value
Gender, n (%)	
Male	30 (60%)
Female	20 (40%)
Age Group	
Infants (0‐2 y)	9 (18%)
Children (3‐11 y)	30 (60%)
Adolescents (12‐17)	11 (22%)
Age, aggregate (y; mean ± SD)	8.19 ± 9.31

### 3.1 Efficacy assessments

All primary and secondary scoring assessments (IGA, EASI, BSA, VAS pruritus, CDLQI, FDLQI, and PSQ Problem Area) mean scores were significantly improved from baseline to 2 weeks and from baseline to end of study (6 weeks) (Table 2).

**Table 2:** Primary and secondary end point scores with mean relative reductions from baseline to 2 and to 6 wk

	Baseline	2 wk	6 wk	Baseline to 2 wk		Baseline to 6 wk	
	Score			Mean relative reduction (%)	*P*‐value	Mean relative reduction (%)	*P*‐value
Primary end points							
EASI	13.8	8.4	6.8	34.2	0.00001	46.0	0.00001
BSA	30.6%	23.2%	19.5%	21.8	0.0006	33.6	0.00001
IGA	3.6	2.7	2.3	23.1	0.00001	35.7	0.00001
Secondary end points							
VAS	6.3	4	3.6	29.0	0.00001	39.1	0.00001
CDLQI	10.5	5.7	5.2	39.8	0.00001	37.7	0.00001
FDLQI	11.7	8.7	6.2	19.7	0.00001	45.9	0.00001
PSQ	6.6	4.9	3.9	19.9	0.0001	34.1	0.00001

### 3.2 Bacteriological assessments

All evaluable subjects (N = 50) had a positive lesional skin SA culture at baseline, with 7 (14%) positive for MRSA and 43 (86%) positive for MSSA. Cultures were repeated after 2 weeks of daily cleansing with NaOCl body wash; 25 of the 43 (58%) MSSA+ subjects remained MSSA+, while all 7 MRSA+ subjects remained positive.

### 3.3 Concurrent topical corticosteroid usage

From the subject diaries, among the 43 subjects using a topical corticosteroid at baseline, a progressive subject‐recorded decrease in corticosteroid usage that equaled a reduction from baseline of 3.6% at week 3, 18.2% at week 4, 30.4% at week 5, and 36.5% at week 6/end of study was found.

### 3.4 Participant/Parent satisfaction and preference

At end of study, an additional questionnaire item, jointly completed by both parent and age‐appropriate child, provided subjective opinions from those who previously had used traditional bleach baths as compared to use of the NaOCl body wash (38 of 50 subjects). Of these 38 subjects who had previously used traditional bleach baths, a majority (23 subjects/parents, 61%) preferred the NaOCl body over traditional bleach baths, while 3 subjects/parents (8%) preferred traditional bleach baths and 12 subjects (32%) declared no preference.

### 3.5 Safety assessments

Of the 50 subjects who completed all study visits, 14 subjects reported 19 AEs. Six subjects (12%) had AEs that were assessed as probably related to the study product (transient warmth or burning sensation at the time of application lasting only seconds to minutes), 3 (6%) were assessed as possibly related to the study product (periocular pruritus, eczema herpeticum, and new‐onset pruritus), and 10 (20%) were assessed as unlikely to be related to the study product (diarrhea, folliculitis, pruritus, ankle sprain, influenza, eczema flare, upper respiratory infection, and cough).

## 4 DISCUSSION

As in a smaller open‐label single‐center study^18^ and consistent with results of previous randomized controlled trials of moderate‐to‐severe AD patients using twice‐weekly dilute bleach baths,^9^, ^10^, ^11^, ^13^ daily NaOCl body wash (0.006%) for 12 weeks led to significant improvement in all AD severity measures. Although the open‐label nature of this study is a major limitation to this study given the lack of a vehicle‐controlled cohort and the lack of blinding of subjects/parents and investigators, this limitation is somewhat offset by the extent of improvement observed with the NaOCl body wash being consistent with the results reported in previous randomized controlled trials of moderate‐to‐severe AD patients using twice‐weekly dilute bleach baths.^9^, ^10^, ^11^, ^13^ A further limitation may include the possibility that bleach may alter the skin's microbial diversity more so than eradicating pathogens such as SA. The NaOCl body wash was well tolerated with transient warmth or burning sensation at application site the most common AEs, described in 6% and 12% of subjects, respectively. However, the NaOCl body wash was deemed more convenient than the bleach bath, showing results with 2 minutes of application vs the 10‐minute bleach bath soak. In addition, having a dilute bleach solution in the household may be a safer option to having full‐strength bleach.

As in studies with bleach baths, the NaOCl body wash did not eliminate SA colonization;^9^, ^10^ all patients with MRSA and most with MSSA had these organisms persist by nonquantitative culture. In fact, the original 0.005% concentration of dilute bleach was chosen because of studies suggesting that this concentration was bactericidal for clinical isolates of *S aureus* and other bacteria. However, recent studies have suggested that this range may not be bactericidal on the skin. Topically applied HOCl (hypochlorous acid, which in alkaline aqueous solution is NaOCl) reduced cytokine expression, itch, and clinical inflammation in the NC/Nga mouse model of AD, but at a concentration of 0.05%, approximately 10‐fold that in dilute bleach baths and the 0.006% NaOCl solution.^25^ In addition, concentrations of 0.01%‐0.16% were required to eradicate *S aureus* biofilms in vitro and a concentration of 0.04% was required to reduce bacteria from AD skin in an ex vivo model.^26^ Most recently, NaOCl 0.005% was found to have no inhibitory effect on the Agr quorum‐sensing system of *S aureus* (which regulates expression of toxins).^27^ The latter study also found no inhibitory effect on two strains of the commensal bacterium, *S epidermidis,* 27 suggesting the possibility for the use of both dilute NaOCl and commensal bacterial as topical therapy in the future.

These studies suggest that the ameliorative effect of the dilute bleach baths and NaOCl wash involves a mechanism beyond its oxidative capability and bactericidal activity against SA.^28^, ^29^ Indeed, Leung et al^30^ have shown that NaOCl has a direct antiinflammatory effect, suppressing NFκB signaling in cultured keratinocytes and reducing the severity of radiation dermatitis in mouse skin. A preliminary report suggested that NaOCl improves skin barrier function and alters inflammatory mediator expression in AD without affecting bacterial dysbiosis.^31^

## 5 CONCLUSIONS

The NaOCl body wash significantly improved all primary and secondary outcome measures, reduced the use of topical corticosteroids, and was well tolerated in children with moderate‐to‐severe AD, supporting its utility as a practical alternative to traditional bleach baths. A key advantage of the NaOCl body wash is convenience, which would be expected to improve adherence for long‐term maintenance care. Additional exploration of the mechanism by which dilute topical NaOCl ameliorates AD is warranted.

## CONFLICTS OF INTEREST

Northwestern University and The UTHealth McGovern Medical School‐Houston received financial support and clinical supplies (commercially available study product) from TopMD, Inc., the makers of CLn^®^ BodyWash, for the clinical trial.
